# SleepNow – A combined cognitive behavioral therapy for insomnia and physical exercise intervention in men with metastatic prostate cancer: results from a feasibility randomized controlled trial

**DOI:** 10.2340/1651-226X.2025.42246

**Published:** 2025-02-09

**Authors:** Beverley Lim Høeg, Katrine Bjerre Løppenthin, Josée Savard, Christoffer Johansen, Jesper Frank Christensen, Mads Nordahl Svendsen, Niels Holländer, Pernille Envold Bidstrup

**Affiliations:** a Psychological Aspects of Cancer, Cancer Survivorship, Danish Cancer Institute, Copenhagen, Denmark; b Phase 1 Unit, Department of Oncology, Copenhagen University Hospital, Copenhagen, Denmark; c School of Psychology, Faculty of Social Sciences, Université Laval and CHU de Québec-Université Laval Research Center, Québec, Canada; d CASTLE, Department of Oncology, Copenhagen University Hospital, Copenhagen, Denmark; e Center for Physical Activity Research, Copenhagen University Hospital, Denmark; f Department of Clinical Oncology and Palliative Care, Zealand University Hospital, Næstved, Denmark; g Department of Psychology, Copenhagen University, Copenhagen, Denmark

**Keywords:** Insomnia, exercise, metastatic cancer, cognitive behavioral therapy, prostate cancer

## Abstract

**Background:**

About 60% of patients with cancer experience sleep disturbances, but few studies have focused on patients living with metastatic cancer. We examined the feasibility of the SleepNow intervention combining cognitive behavioral therapy for insomnia (CBT-I) with physical exercise in men with metastatic prostate cancer (mPCa).

**Patients/material and methods:**

We conducted a feasibility randomized trial in patients under treatment for castration resistant mPCa with insomnia (Insomnia Severity Index [ISI] score ≥ 8). Patients were randomized 1:1 to either SleepNow or usual care. SleepNow is a manualized 12-week program consisting of bi-weekly sessions of physical exercise and four nurse-led sessions of CBT-I. Patients in usual care received no insomnia treatment. We assessed feasibility and measured objective and patient-reported outcomes at baseline and 3-months follow-up. Changes in both groups were compared using the Wilcoxon test.

**Results:**

We randomized 12 patients (5 intervention and 7 control; age range = 59–81 years, mean Gleason score = 7.75, mean time since diagnosis ≈ 7 years). Intervention patients reported high satisfaction, all attended at least three CBT-I sessions (75%) and four completed at least 20 of the 24 training sessions. The intervention group showed improvements in insomnia, sleep quality, fatigue, anxiety, depression and health-related quality-of-life but between-group differences were not statistically significant.

**Interpretation:**

The SleepNow intervention is the first to combine nurse-delivered CBT-I and physical exercise and was acceptable and potentially efficacious. Our results are important for targeting sleep interventions to the growing population of patients living long term with metastatic cancer.

## Introduction

Insomnia is a frequent problem in patients with cancer and about 60% of patients experience sleep difficulties throughout the disease trajectory, including those living with advanced stage/metastatic cancer [1, 2]. Chronic sleep problems affect well-being, as well as inflammatory, cardiovascular and metabolic processes that may increase the risk of comorbidity and even premature death [3, 4]. Often precipitated by the stresses of diagnosis and the side-effects of treatment, insomnia may become chronic due to factors like behaviors and thought patterns that perpetuate poor sleep [5, 6]. Likewise, physiological factors such as the hormonal regulation of the body’s temperature and circadian rhythm disruptions may also perpetuate insomnia [7] and these might be especially important in patients undergoing long-term hormonal treatment [8]. Men living with metastatic prostate cancer (mPCa) is a large patient population, where the mainstay of treatment is androgen-deprivation therapy (ADT), which has been shown to increase the risk of sleep difficulties [9, 10].

As sleep medications have undesirable side-effects [11], and patients often refuse drugs for fear that it might interfere with their cancer treatment [6], it is important to develop and offer non-pharmacological treatment options for insomnia. Current guidelines recommend cognitive behavioral therapy for insomnia (CBT-I) as first line treatment [12], but physical exercise is also recommended [12], as it addresses the physiological mechanisms of sleep through, for example, improved thermoregulation, increased melatonin and regulation of endorphins, serotonin and kynurenine [13–15]. However, there is a lack of evidence supporting the efficacy of exercise as a stand-alone treatment for insomnia [12]. Furthermore, the evidence base for both CBT-I and exercise consists primarily of trials carried out in cured breast cancer survivors, indicating a lack of interventions developed for patients with metastatic cancer, especially men [16, 17].

Taking all the above into account, a combination of CBT-I focusing on maladaptive sleep behaviors and cognitions, and physical exercise addressing physiological mechanisms, may potentially be superior to single interventions in the population of patients living with metastatic cancer, and in particular, men with mPCa. To our knowledge, this has yet to be developed and tested. Given the investment required to carry out a full trial of a new intervention, it is important to first assess whether an intervention is feasible and acceptable [18]. The aims of this study were to: (1) develop the SleepNow intervention combining nurse-led CBT-I and structured physical exercise, and (2) test the feasibility of SleepNow in men with mPCa with insomnia.

## Patients/materials and methods

### Study design

This was a feasibility randomized controlled trial reported according to The Consolidated Standards of Reporting Trials (CONSORT) statement extension for randomized feasibility studies [19]. Between February and September 2018, three project nurses consecutively identified potentially eligible patients attending outpatient treatment at the oncology department of Zealand University Hospital, Næstved, Denmark. Inclusion criteria were: ≥ 18 years with histologically confirmed castration-resistant mPCa, receiving ADT (enzalutamide or abiraterone) alone or in combination with chemotherapy (docetaxel and cabazitaxel) and able to speak and write Danish. Patients with cognitive/psychiatric impairment, inability to participate in an exercise program, who worked night shifts, were already exercising more than three times per week or had no sleep difficulties, were excluded. We aimed to include 20–24 patients [20].

Patients reporting sleep problems and who were interested in the study were screened by nurses for insomnia using the Insomnia Severity Index (ISI). Patients scoring ≥8 were randomized 1:1 upon written informed consent to either the SleepNow intervention group (CBT-I and exercise) or to the control group by a project nurse using a computer-generated algorithm. Blinding was not possible after allocation. Patients in the control group received usual care, that is, no behavioral intervention for insomnia. This study was approved by the Ethics Committee of the Capital Region of Denmark (No. H-17021054), the Health and Medical Research Committee of Region Zealand (REG-081-2017) and the Danish Data Protection Agency (Jr.no.: 2012-58-0003).

### The SleepNow intervention

SleepNow is a manualized 12-week program consisting of four sessions of nurse-led CBT-I conducted every third week and 24 bi-weekly individual sessions of physical exercise supervised by a physiotherapist.

The four individual 60-min CBT-I sessions included psychoeducation on sleep and the benefits of exercise, and behavioral strategies to improve sleep (Supplementary Table 1). We adapted Savard et al.’s psychologist-led CBT-I program that has been shown to be effective in cancer populations [21]. Project nurses attended a 4-day educational program that included theoretical background on sleep and insomnia, training in behavioral change and cognitive strategies and an overview of the exercise/training components in the intervention. No formal assessment of fidelity to the manual was carried out, but informal supervision was provided by the study coordinator on a weekly basis.

The exercise intervention consisted of 2 weekly 60–90 min training sessions for 12 weeks that took place at the hospital’s rehabilitation center (Supplementary Table 2). The program followed current exercise recommendations to improve endurance and contained a 40–60-min period of moderate aerobic exercise on stationary bicycles, followed by a shorter period of strength training of the lower extremities. Exercise intensity was individualized for each patient based on a test of cardiovascular function (Wattmax test) and muscle strength (1 RM test) at baseline and patients progressed to higher intensities over the course of the 12 weeks. For each session, the physiotherapist recorded information on the duration and intensity of the aerobic and strength training exercises and whether any modifications were made.

### Data collection

We collected objective and patient-reported questionnaire data at baseline before randomization and again at 3 months follow-up or after the completion of the intervention. Nurses and physiotherapists also recorded any adverse events. Feasibility outcomes included recruitment, acceptability (assessed by patients’ adherence and satisfaction with the intervention), as well as motivation and barriers to participation. These were extracted from recruitment and attendance logs, and from interviews at baseline and follow-up. Objective outcomes consisted of the patient’s cardiorespiratory fitness assessed using a progressive cycle ergometer test and reported as the maximum aerobic capacity of the patient (VO2max) [22], while sleep was measured through an actigraphy device worn on the patient’s wrist for 7 days at baseline and at follow-up. Patient-reported outcomes included validated measures of insomnia symptoms, sleep quality, physical activity, fatigue, perceived stress, anxiety, depression and health-related quality of life (HRQoL) (Supplementary Table 3). Patients in the control group were asked to fill in a sleep diary to track daily bed and wake times, as well as use of sleep medication if any, for 7 days at baseline and again at follow-up, while the intervention group was asked to fill in a sleep diary for all 12 weeks.

### Data analysis

In accordance with CONSORT guidelines for feasibility studies, we describe our data and assessed outcomes using standard descriptive methods [19]. We present baseline and follow-up results for the intervention and control groups. Where possible, we handled any missing responses using the scoring guidelines for missing data for that measure. For all other cases, analysis was based on complete cases. To assess potential efficacy of the intervention, we compared changes from baseline to follow-up in the intervention group to the control group using the non-parametric Wilcoxon test.

### Patient involvement in research

During the development phase of this study, we carried out a focus group with a panel of five men with mPCa where we presented the study and received feedback on the patient materials, the intervention and the questionnaires. Based on the feedback, we revised the patient materials and treatment manual to improve clarity and relevance.

## Results

**Figure 1:**
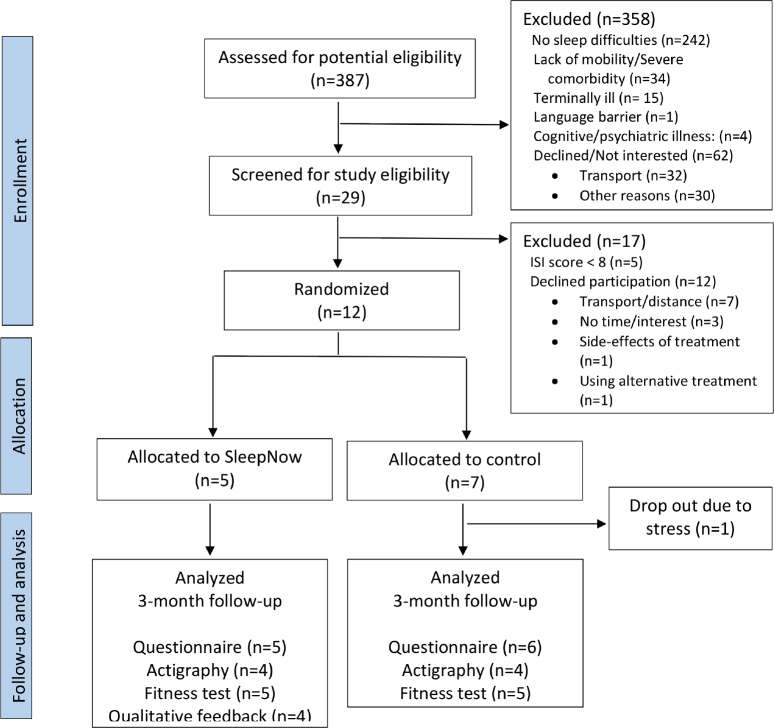
Flow diagram of the SleepNow randomized feasibility study.

A total of 387 patients were evaluated for eligibility, 29 patients consented to be screened for insomnia and 24 patients met inclusion criteria (ISI score ≥ 8), of which 12 consented to participate and were randomized: five to the intervention group and seven to the control group (Figure 1). We were thus unable to reach the planned number of 20 patients within the recruitment period. Patients were generally above 70 years, had only a high-school education (67%) and had on average been living with prostate cancer for approximately 7 years prior to inclusion (Table 1). No serious adverse events affecting patient safety were reported.

**Table 1:** Baseline characteristics of the study sample.

Measures	Intervention *N* = 5	Control *N* = 7
**Sociodemographic factors**		
Age (years)^a^	75 (71–81)	70 (59–77)
Cohabiting^b^ Live alone Live with partner	1 4	1 6
Highest attained education^b^ Primary/High school Short to middle higher education Long higher education (university)	3 2 0	5 2 0
Occupation^b^ Unemployed Employed Age-related retirement	0 0 5	2 2 3
**Lifestyle/behavioral factors**		
Alcohol^a^ No. of units per week None	4.6 (1–10) 0	6.6 (1–20) 1
Smoking^b^ Current Former Never smoked	1 3 1	0 7 0
Caffeine^b^ Less than 5 cups per day 5 or more cups per day	3 2	5 2
BMI^a^		
Duration of sleep problems (years)^a^	3 (1–5)	12.5 (3–50)
Clinical information		
Time since diagnosis (years)^a^	7.75 (4–12)	6.80 (3–12)
Gleason score^a^	7.75 (7–9)	7.75 (7–8)
Prostate-specific antigen (PSA)^a^	21.6 (9–48.8)	7.80 (7–8)

a mean (range),

b number

### Adherence and acceptability

Four out of the five men in the intervention group completed at least 80% of the exercise program (20 out of 24 sessions) and all patients attended at least three CBT-I sessions (75%). Feedback showed that a strong motivation for participating was the desire to address their sleep problems without the use of medications and wanting to take action to ‘improve my health’. The opportunity to exercise with others was also stated as a motivator, as well as the supervision from a physiotherapist. Barriers reported included the travel distance to the training center and the time commitment needed for the exercise sessions.

### Objective and patient-reported outcomes

Across groups, patients experienced poor objective sleep at baseline, with a high number of awakenings at night and sleep efficiencies below the normal 85% (Table 2). At follow-up, patients in the intervention group showed poorer objective sleep, but self-reported improvements on a range outcomes including insomnia, sleep quality, fatigue, pain, anxiety, depression and HRQoL (Table 3). However, differences between the intervention group and control group were not statistically significant on either objective or patient-reported outcomes. There was poor adherence to filling out the sleep diary with large amounts of missing data regarding daily bed and wake times and we were unable to report on these results.

**Table 2:** Changes in sleep (actigraphy) and fitness (physical test) according to intervention and control group.

Measure	Intervention (*n* = 5)			Control (*n* = 7)			Difference between groups p*
	Baseline Mean (SD)	Follow-up Mean (SD)	Difference Mean (SD)	Baseline Mean (SD)	Follow-up Mean (SD)	Difference Mean (SD)	
**Actigraphy**	*n* = 4^a^	*n* = 5		*n* = 7	*n* = 4^b^		
Total sleep (minutes)	429.0 (53.0)	420.2 (61.0)	–17.8 (16.5)	414.0 (65.4)	474.5 (69.7)	46.8 (14.4)	0.07
Sleep onset latency (minutes)	26.8 (7.2)	29.5 (4.3)	1.5 (4.0)	32.3 (17.9)	39.3 (30.2)	5.1 (14.5)	0.89
Sleep efficiency (%)	79.3 (4.8)	79.3 (5.4)	–1.1 (3.7)	76.3 (5.6)	75.9 (5.5)	1.8 (2.7)	0.35
WASO (minutes)	79.9 (24.2)	67.1 (33.3)	–8.3 (26.5)	71.9 (20.9)	93.7 (25.3)	7.6 (22.3)	0.49
No. of awakenings	71.1 (15.7)	69.0 (32.6)	0.4 (22.4)	75.8 (29.5)	106.6 (46.5)	15.8 (15.6)	0.24
**Fitness test**	*n* = 5	*n* = 5		*n* = 6^c^	*n* = 5^d^		
Oxygen consumption (l/min)	1.6 (0.1)	1.6 (0.1)	–0.1 (0.1)	1.6 (0.2)	1.5 (0.2)	0.0 (0.1)	1.00
Relative VO2max (mL/min/kg)	19.1 (2.8)	18.2 (2.5)	–0.9 (1.2)	18.2 (3.1)	16.7 (2.9)	–0.5 (1.5)	0.84

* *p*-values from a Wilcoxon test for two independent samples.

a The actigraph of one intervention patient malfunctioned and his data was not recorded.

b Two patients in the control group dropped out and a third patient was hospitalized during the time period when he had to wear the actigraph, yielding invalid data.

c We were unable to calculate results for one patient due to a recording error.

d Two patients in the control group dropped out.

**Table 3:** Changes in patient-reported outcomes from baseline to 3 months follow-up according to intervention and control group.

Measure (Score range)	Intervention group (*n* = 5)			Control group (*n* = 7)			Differences between groups p*
	Baseline Mean (SD)	Follow-up Mean (SD)	Difference Mean (SD)	Baseline Mean (SD)	Follow-up Mean (SD)	Difference Mean (SD)	
**Insomnia Severity Index (ISI) (0**–**28)**	*n* = 5 14.0 (4.4)	*n* = 5 8.8 (1.6)	-5.2 (4.4)	*n* = 7 20.3 (3.4)	*n* = 6 19.2 (6.8)	–0.7 (3.9)	0.08
**Pittsburgh Sleep Quality Index (PSQI)**	*n* = 5	*n* = 5		*n* = 7	*n* = 6		
Global score (0–21)	10.2 (4.2)	8 (2.9)	–2.2 (2.3)	12.9 (1.3)	12.5 (3.9)	–0.5 (2.7)	0.38
Sleep quality (0–3)	1.6 (0.6)	1.00 (0.7)	–0.6 (0.6)	2.1 (0.7)	2.3 (0.8)	0.2 (0.4)	0.07
Sleep latency (0–3)	1.4 (1.1)	1.2 (1.1)	–0.2 (0.5)	1.7 (1.0)	1.7 (1.4)	0 (0.6)	0.65
Sleep duration (0–3)	1.8 (1.1)	1 (0.7)	–0.8 (1.3)	1.7 (1.0)	1.7 (1.4)	0 (0.6)	0.31
Sleep efficiency (0–3)	2.6 (0.9)	1.6 (0.9)	–1.0 (1.0)	2.4 (0.8)	2.3 (0.8)	0 (0.6)	0.14
Sleep disturbances (0–3)	1.2 (0.5)	1.4 (0.6)	0.2 (0.5)	1.7 (0.5)	1.5 (0.6)	–0.3 (0.5)	0.16
Use of sleep medication (0–3)	0.6 (1.3)	0.6 (1.3)	0 (0)	1.6 (1.5)	1.5 (1.6)	–0.3 (1.4)	1.00
Daytime dysfunction (0–3)	1.2 (0.84)	1.2 (0.45)	0 (1)	1.6 (0.8)	1.5 (0.8)	0 (1.1)	1.00
**Multidimensional Fatigue Inventory (MFI)**							
General fatigue (4–20)	*n* = 4 13.8 (1.0)	*n* = 5 11.2 (0.5)	–2.5 (1.3)	*n* = 7 15.1 (3.7)	*n* = 6 15.5 (3.5)	1.2 (2.7)	0.10
Physical fatigue (4–20)	*n* = 4 12.0 (2.2)	*n* = 5 12.8 (4.6)	–0.8 (2.8)	*n* = 7 12.9 (5.2)	*n* = 6 12.8 (3.7)	–1.3 (3.3)	0.74
Reduced activity (4–20)	*n* = 4 12.8 (0.2)	*n* = 5 13.0 (4.1)	–1.3 (2.2)	*n* = 7 15.0 (3.9)	*n* = 6 14.7 (4.4)	0 (1.9)	0.47
Reduced motivation (4–20)	*n* = 4 11.3 (2.4)	*n* = 5 9.0 (2.7)	–2.0 (1.2)	*n* = 7 8.7 (2.4)	*n* = 6 8.0 (3.4)	–0.2 (4.6)	0.47
Mental fatigue (4–20)	*n* = 5 9.0 (3.9)	*n* = 5 8.8 (2.5)	–0.2 (2.7)	*n* = 7 12.3 (4.1)	*n* = 6 12.5 (5.4)	0.5 (2.4)	0.64
**GAD-7 (0**–**21)**	*n* = 5 5.4 (5.9)	*n* = 5 2.2 (2.1)	–3.2 (5.0)	*n* = 7 8.3 (6.3)	*n* = 6 9.0 (7.3)	1.8 (5.6)	0.59
**PHQ-9 (0**–**27)**	*n* = 5 6.4 (5.4)	*n* = 5 3.6 (1.1)	–2.8 (4.4)	*n* = 7 11.0 (6.4)	*n* = 6 10.8 (5.6)	0.5 (6.8)	0.26
**Perceived Stress Scale (PSS) (0**–**40)**	*n* = 5 14.8 (7.1)	*n* = 5 13.2 (6.3)	–1.6 (3.4)	*n* = 7 15.1 (9.3)	*n* = 6 14.0 (7.0)	1.8 (6.4)	0.34
**EORTC-C30 (0**–**100)**							
Global health status/QoL	*n* = 5 63.33 (21.73)	*n* = 5 70.00 (15.14)	6.67 (20.75)	*n* = 7 53.57 (21.97)	*n* = 6 47.22 (31.91)	–5.56 (32.35)	0.43
Physical functioning/PF	*n* = 4 78.33 (16.67)	*n* = 5 77.33 (19.78)	0 (21.77)	*n* = 7 72.38 (27.06)	*n* = 6 58.89 (29.64)	–10.00 (17.26)	0.60
Role functioning/RF	*n* = 4 79.17 (25.00)	*n* = 5 83.33 (16.67)	0 (25.57)	*n* = 7 64.29 (45.57)	*n* = 6 61.11 (47.92)	2.77 (24.53)	0.83
Emotional functioning/EF	n = 4 75.00 (31.91)	*n* = 5 83.33 (16.67)	8.33 (16.67)	*n* = 6 66.67 (26.35)	*n* = 5 75.00 (19.54)	–1.67 (21.57)	0.62
Cognitive functioning/CF	*n* = 4 83.33 (33.33)	*n* = 5 86.67 (18.26)	8.33 (16.67)	*n* = 7 64.28 (24.40)	*n* = 6 66.67 (33.33)	0 (23.57)	0.58
Social functioning/SF	*n* = 4 70.83 (28.46)	*n* = 5 80.00 (21.73)	4.17 (15.96)	*n* = 7 52.38 (17.82)	*n* = 6 75.00 (29.34)	22.22 (20.18)	0.22
Fatigue/FA	*n* = 4 38.89 (19.24)	*n* = 5 35.56 (19.88)	0 (15.71)	*n* = 7 55.56 (19.25)	*n* = 6 71.11 (33.33)	15.56 (21.66)	0.42
Nausea and vomiting/NV	*n* = 4 0 (3.33)	*n* = 5 3.33 (7.45)	4.17 (8.33)	*n* = 7 4.76 (8.13)	*n* = 6 22.22 (25.09)	16.67 (25.82)	0.48
Pain/PA	*n* = 4 41.67 (34.69)	*n* = 5 13.33 (21.73)	–25 (21.52)	*n* = 5 50.00 (50.00)	*n* = 5 53.33 (38.00)	3.33 (32.06)	0.21
Dyspnoea/DY	*n* = 4 8.33 (16.67)	*n* = 5 13.33 (18.56)	0 (27.22)	*n* = 7 33.33 (38.49)	*n* = 6 38.89 (49.07)	0 (21.08)	1.00
Insomnia/SL	*n* = 4 66.67 (27.22)	*n* = 5 33.33 (23.57)	-33.33 (27.22)	*n* = 7 80.95 (26.23)	*n* = 6 83.33 (18.26)	5.56 (25.09)	0.16
**Physical and everyday activity (study specific items)**							
1) physical activity (1–5)	*n* = 5 1.4 (0.89)	*n* = 5 2.6 (1.14)	1.2 (1.3)	*n* = 7 3.29 (1.25)	*n* = 6 2.5 (1.38)	–0.5 (1.38)	0.14
< 60 min/week	5 (100%)	4 (80%)		4 (57%)	5 (83%)		
> 60 min/week	0	1 (20%)		3 (43%)	1 (17%)		
2) everyday activity (1–7)	2.8 (1.10)	3.2 (1.64)	0.4 (2.3)	4.57 (1.81)	4.17 (1.47)	0 (1.67)	0.85
< 60 min/week	3 (60%)	4 (80%)		3 (43)	3 (50%)		
> 60 min/week	2 (40%)	1 (20%)		4 (57)	3 (50%)		
**International Physical Activity Questionnaire – short form (IPAQ)**							
Vigorous intensity/days a week	*n* = 5 0.8 (1.30)	*n* = 5 1.2 (1.30)	0.4 (2.07)	*n* = 6 2.5 (2.59)	*n* = 5 2.0 (1.87)	–1.25 (3.20)	0.72
Vigorous intensity/minutes per day	*n* = 1 90 (0)	*n* = 1 120 (0)	*n*/a	*n* = 4 75 (30)	*n* = 3 60 (0)	0 (0)	n/a
Moderate intensity/days a week	*n* = 5 2.2 (1.10)	*n* = 5 1.6 (2.1)	–0.6 (2.07)	*n* = 4 1.88 (1.65)	*n* = 6 1.83 (1.83)	0.17 (0.29)	0.77
Moderate intensity/minutes per day	*n* = 4 105 (62.44)	*n* = 2 60 (0)	30 (.)	*n* = 4 112.5 (86.17)	*n* = 2 75 (21.21)	0 (0)	0.55
Walking/days per week	*n* = 4 2.25 (2.63)	*n* = 5 4.4 (2.7)	2.75 (2.87)	*n* = 6 6.17 (2.04)	*n* = 5 4.6 (2.88)	–1.4 (2.6)	0.04
Walking/minutes per day	*n* = 2 65 (35.36)	*n* = 3 130 (147.99)	115 (134.35)	*n* = 4 65 (76.81)	*n* = 3 25 (18.03)	–1.67 (23.6)	0.44
Sitting/minutes per day	*n* = 4 352.5 (166.81)	*n* = 4 300 (84.85)	–90 (137.48)	*n* = 4 330 (158.75)	*n* = 3 520 (91.65)	120 (158.7)	0.25
**EQ-5D-5L**	*n* = 4	*n* = 5		*n* = 7	*n* = 6		
Mobility (1–5)	2.0 (0.82)	1.8 (1.10)	0 (0.82)	1.86 (1.21)	1.83 (1.33)	–0.17 (0.98)	1.00
Self-care (1–5)	1.25 (0.50)	1.2 (0.45)	0 (0)	1.0 (0)	1.33 (0.82)	0.33 (0.82)	0.56
Daily activities (1–5)	2.25 (0.5)	1.4 (0.55)	–0.75 (0.5)	2.43 (1.51)	2.50 (1.52)	0 (1.26)	0.34
Pain/discomfort (1–5)	2.25 (1.26)	1.6 (0.89)	–0.5 (0.58)	2.29 (1.11)	2.5 (1.05)	0.17 (0.41)	0.12
Anxiety/depression (1–5)	1.75 (0.96)	0.4 (0.55)	–0.5 (0.58)	2.0 (1.15)	2.33 (1.63)	0.67 (1.75)	0.24

* *p*-values from a Wilcoxon test for two independent samples.

## Discussion

The SleepNow intervention demonstrated promising acceptability and patients reported high satisfaction and adherence. It is the first insomnia intervention combining nurse-delivered CBT-I and physical exercise for male patients and those living with metastatic cancer, populations that have been understudied in insomnia research. Although our sample size as a feasibility study was not powered to detect statistically significant between-group differences in outcomes, only the intervention group reported improvements on insomnia, fatigue, anxiety, depression and quality of life, suggesting the potential efficacy of SleepNow. The lack of improvements on the objective measures in our study is a common finding in previous studies [23], and may not be a limitation as insomnia and its impact on quality of life is primarily a subjective matter [24].

A future RCT is needed to ascertain whether a combined intervention is more effective than CBT-I alone. However, as insomnia in patients receiving long-term hormonal treatment may be due to physiological factors related to treatment (e.g. dysfunctional thermoregulation and dysregulated circadian rhythms)[7], physical exercise may be a valuable additional component as it targets multiple physiological mechanisms and helps synchronize the circadian rhythm that is essential for a healthy sleep cycle [25]. Our study shows that even older patients with lower fitness levels due to ongoing cancer treatment may successfully complete a tailored exercise program, and that the opportunity to be supervised and to exercise with other patients was found to be valuable. Future studies may furthermore assess whether online formats of CBT-I and exercise training may be viable options for patients with metastatic cancer, as has been shown with cured cancer survivors [26, 27].

A strength of this study was the involvement of patients in the development of study materials and adaption of study procedures to improve patient relevance. In line with recommendations in sleep research, we also collected both subjective and objective measures of sleep using actigraphy [23]. We focused exclusively on patients living with metastatic cancer and as our hospital serves an older population with lower socioeconomic positions, our results have higher generalizability compared to trials that mainly include high-resource patients. Limitations include our small sample size and missing data, for example from the sleep diaries. A future randomized controlled trial may include automatic electronic reminders (e.g. by short text messages) to improve adherence to the sleep diaries and validate the reliability of the objective sleep metrics [28].

Our modified version of CBT-I may enable more cost-effective delivery in hospitals or rehabilitation settings where psychologists are not available, while still delivering the same effective treatment components [21]. Offering a ‘minimum effective dose’ may also be less burdensome for older and more ill patients, and brief versions of CBT-I were found to be non-inferior to traditional eight-session CBT-I programs in non-cancer populations [29].

## Conclusion

The SleepNow intervention was acceptable and potentially efficacious in men with mPCa. We need sleep interventions targeting the growing population of patients living long term with metastatic cancer, given the potential negative consequences of poor sleep on HRQoL, treatment adherence and, possibly, survival.

## Supplementary Material

SleepNow – A combined cognitive behavioral therapy for insomnia and physical exercise intervention in men with metastatic prostate cancer: results from a feasibility randomized controlled trial

## Data Availability

The participants of this study did not give written consent for their data to be shared publicly, so due to the nature of the research data is not available. However, we welcome research collaborations.
