# Supplementary material for: SleepNow – A combined cognitive behavioral therapy for insomnia and physical exercise intervention in men with metastatic prostate cancer: results from a feasibility randomized controlled trial
Source: Acta Oncol. 2025 Feb 9;64:42246. doi: 10.2340/1651-226X.2025.42246 (PMC11833330; doi:10.2340/1651-226X.2025.42246)
Supplement: SleepNow – A combined cognitive behavioral therapy for insomnia and physical exercise intervention in men with metastatic prostate cancer: results from a feasibility randomized controlled trial [file AO-64-42246-s1.pdf]

Supplementary material has been published as submitted. It has not been copyedited, or typeset by Acta Oncologica

SUPPLEMENTARY MATERIALS

Supplementary Table 1. Overview of the Cognitive Behavioral Therapy for Insomnia (CBT-I) program.

Session	Content
Session 1	**Psychoeducation**<br>• Knowledge about sleep, the sleep cycle, insomnia and how insomnia can evolve into a chronic problem<br>• Introduction to the self-management approach, strategies and maladaptive behaviors that perpetuate difficulties initiating or maintaining sleep<br>• The positive effects of physical exercise on sleep<br>• Review of the sleep diary
Session 2	**Stimulus control and sleep restriction**<br>• Introduction of behavioral strategies<br>• Stimulus control: re-associate the bed and the bedroom environment with sleep; create conditions that facilitate sleep; establish a regular sleep-wake rhythm<br>• Sleep restriction: curtail the time in bed to the actual sleep time, thereby creating mild sleep deprivation and resulting in more consolidated and more efficient sleep<br>• Review of the sleep diary and revision of the sleep window for the next week if appropriate<br>• Homework: apply the behavioral strategies
Session 3	**Cognitive management & sleep hygiene**<br>• Introduction to negative thought patterns and how they affect sleep<br>• Cognitive restructuring: identifying and reframing unhelpful thoughts related to anxiety, sleep and physical exercise<br>• Sleep hygiene: habits and environmental factors that promote sleep, e.g., comfortable bedroom temperature, avoiding caffeine, etc.<br>• Review of the sleep diary and revision of the sleep window for the next week if appropriate<br>• Homework: continue application of behavioral strategies and practice cognitive restructuring
Session 4	**Evaluation of progress and relapse prevention**<br>• Review of the sleep diary and revision of the sleep window for the next week if appropriate<br>• Troubleshooting difficulties applying the behavioral and cognitive strategies and how to maintain progress<br>• Elicit patients feedback on the session and the overall program

Supplementary Table 2. Content and progression of the exercise training program.

Week	Content
Week 1-4	Aerobic exercise: cycling 40 minutes at 45-55 % wattmax (target Heart Rate (HR) zone: 60-80% of HR max) (including 5-10 minutes warm up and 5 minutes cool down).<br><br>Strength training: leg pressure three set of 70% of 1 RM until failure (max 15 repetitions) and with 3 minutes break between each set.
Week 5-8	Aerobic exercise: cycling 50 minutes at 50-60 % wattmax (target HR 70-90 % of HR max) (including 5-10 minutes warm up and 5 minutes cool down).<br><br>Strength training: leg pressure four set of 70 % of 1 RM until failure (max 15 repetitions) and with 3 minutes break between each set.
Week 9-12	Aerobic exercise: cycling 60 minutes at 50-60 % wattmax (targeted HR 70-90% of HR max) (including 5-10 minutes warm up and 5 minutes cool down).<br><br>Strength training: leg pressure five set of 70 % of 1 RM until failure (max 15 repetitions) and 3 minutes break between each set.

Supplementary Table 3. List of outcome measures used in the questionnaires

OUTCOME	MEASURE	DESCRIPTION
Insomnia	Insomnia Severity Index (ISI)[1]	Range 0-28; higher scores = worse insomnia
Sleep	Pittsburg Sleep Quality Index (PSQI)[2]	Global score range 0-21 & subscales score range 0-3; higher scores = worse sleep
Fatigue	Multidimensional Fatigue Inventory (MFI)[3]	Range 4-20; higher scores = worse fatigue
Anxiety	Generalized Anxiety Disorder-7 (GAD-7)[4]	Range 0-21; higher scores = more symptoms
Depression	Patient Health Questionnaire-9 (PHQ-9)[5]	Range 0-27; higher scores = more symptoms
Stress	Perceived Stress Scale (PSS-10)[6]	Range 0-40; higher scores = higher perceived stress
Physical activity (PA)	International Physical Activity Questionnaire - short form (IPAQ)[7]	Scored as days per week and minutes per day for vigorous PA, moderate PA, walking and sitting
Physical and everyday activity	Two study specific items assessing number of minutes per week	Answered on the following scale:<br>1 = 0 min<br>2 = < 30 min<br>3 = 30-60 min<br>4 = 60-90 min<br>5 = 90-150 min<br>6 = 160-300 min<br>7 = > 300 min
Health-related quality of life (HRQoL)	European Organisation for Research and Treatment of Cancer – Core questionnaire (EORTC-C30)[8]	Range 0-100;<br>For functioning subscales: higher scores = better HRQoL<br>For symptom subscales: higher scores = more symptoms

	EuroQol (EQ-5D-5L)[9]	In this study, we used single item score range 1-5; Higher scores = worse outcome

REFERENCES

1 Bastien, C. H., Vallières, A. & Morin, C. M. Validation of the Insomnia Severity Index as an outcome measure for insomnia research. Sleep Med. 2, 297-307 (2001). https://doi.org:10.1016/s1389-9457(00)00065-4

2 Buysse, D. J., Reynolds, C. F., 3rd, Monk, T. H., Berman, S. R. & Kupfer, D. J. The Pittsburgh Sleep Quality Index: a new instrument for psychiatric practice and research. Psychiatry Res. 28, 193-213 (1989). https://doi.org:10.1016/0165-1781(89)90047-4

3 Smets, E. M., Garssen, B., Bonke, B. & De Haes, J. C. The Multidimensional Fatigue Inventory (MFI) psychometric qualities of an instrument to assess fatigue. J. Psychosom. Res. 39, 315-325 (1995). https://doi.org:10.1016/0022-3999(94)00125-o

4 Spitzer, R. L., Kroenke, K., Williams, J. B. & Lowe, B. A brief measure for assessing generalized anxiety disorder: the GAD-7. Arch. Intern. Med. 166, 1092-1097 (2006). https://doi.org:10.1001/archinte.166.10.1092

5 Kroenke, K., Spitzer, R. L. & Williams, J. B. The PHQ-9: validity of a brief depression severity measure. J. Gen. Intern. Med. 16, 606-613 (2001). https://doi.org:10.1046/j.1525-1497.2001.016009606.x

6 Cohen, S., Kamarck, T. & Mermelstein, R. A Global Measure of Perceived Stress. J. Health Soc. Behav. 24, 385-396 (1983). https://doi.org:10.2307/2136404

7 Craig, C. L. et al. International physical activity questionnaire: 12-country reliability and validity. Med. Sci. Sports Exerc. 35, 1381-1395 (2003). https://doi.org:10.1249/01.Mss.0000078924.61453.Fb

8 Aaronson, N. K. et al. The European Organization for Research and Treatment of Cancer QLQ-C30: a quality-of-life instrument for use in international clinical trials in oncology. J. Natl. Cancer Inst. 85, 365-376 (1993). **https://doi.org:10.1093/jnci/85.5.365**

9 EuroQol. EuroQol--a new facility for the measurement of health-related quality of life. Health Policy 16, 199-208 (1990). https://doi.org:10.1016/0168-8510(90)90421-9

CONSORT 2010 checklist of information to include when reporting a pilot or feasibility trial*

Section/Topic	Item No	Checklist item	Reported on page No
**Title and abstract**			
	1a	Identification as a pilot or feasibility randomised trial in the title	1
	1b	Structured summary of pilot trial design, methods, results, and conclusions (for specific guidance see CONSORT abstract extension for pilot trials)	2
**Introduction**			
Background and objectives	2a	Scientific background and explanation of rationale for future definitive trial, and reasons for randomised pilot trial	3-4
	2b	Specific objectives or research questions for pilot trial	4
**Methods**			
Trial design	3a	Description of pilot trial design (such as parallel, factorial) including allocation ratio	4
	3b	Important changes to methods after pilot trial commencement (such as eligibility criteria), with reasons	n/a
Participants	4a	Eligibility criteria for participants	4
	4b	Settings and locations where the data were collected	4
	4c	How participants were identified and consented	5
Interventions	5	The interventions for each group with sufficient details to allow replication, including how and when they were actually administered	5-6
Outcomes	6a	Completely defined prespecified assessments or measurements to address each pilot trial objective specified in 2b, including how and when they were assessed	6
	6b	Any changes to pilot trial assessments or measurements after the pilot trial commenced, with reasons	n/a
	6c	If applicable, prespecified criteria used to judge whether, or how, to proceed with future definitive trial	n/a

Sample size	7a	Rationale for numbers in the pilot trial	5
	7b	When applicable, explanation of any interim analyses and stopping guidelines	None pre-planned
Randomisation:			
Sequence generation	8a	Method used to generate the random allocation sequence	5
	8b	Type of randomisation(s); details of any restriction (such as blocking and block size)	5
Allocation concealment mechanism	9	Mechanism used to implement the random allocation sequence (such as sequentially numbered containers), describing any steps taken to conceal the sequence until interventions were assigned	5
Implementation	10	Who generated the random allocation sequence, who enrolled participants, and who assigned participants to interventions	5
Blinding	11a	If done, who was blinded after assignment to interventions (for example, participants, care providers, those assessing outcomes) and how	Blinding not possible
	11b	If relevant, description of the similarity of interventions	5
Statistical methods	12	Methods used to address each pilot trial objective whether qualitative or quantitative	7
**Results**			
Participant flow (a diagram is strongly recommended)	13a	For each group, the numbers of participants who were approached and/or assessed for eligibility, randomly assigned, received intended treatment, and were assessed for each objective	Figure 1
	13b	For each group, losses and exclusions after randomisation, together with reasons	Figure 1
Recruitment	14a	Dates defining the periods of recruitment and follow-up	4
	14b	Why the pilot trial ended or was stopped	8
Baseline data	15	A table showing baseline demographic and clinical characteristics for each group	Table 1
Numbers analysed	16	For each objective, number of participants (denominator) included in each analysis. If relevant, these numbers should be by randomised group	All tables

Outcomes and estimation	17	For each objective, results including expressions of uncertainty (such as 95% confidence interval) for any estimates. If relevant, these results should be by randomised group	All tables
Ancillary analyses	18	Results of any other analyses performed that could be used to inform the future definitive trial	8
Harms	19	All important harms or unintended effects in each group (for specific guidance see CONSORT for harms)	8
	19a	If relevant, other important unintended consequences	n/a
**Discussion**			
Limitations	20	Pilot trial limitations, addressing sources of potential bias and remaining uncertainty about feasibility	10
Generalisability	21	Generalisability (applicability) of pilot trial methods and findings to future definitive trial and other studies	10
Interpretation	22	Interpretation consistent with pilot trial objectives and findings, balancing potential benefits and harms, and considering other relevant evidence	9-10
	22a	Implications for progression from pilot to future definitive trial, including any proposed amendments	9-10
**Other information**			
Registration	23	Registration number for pilot trial and name of trial registry	n/a
Protocol	24	Where the pilot trial protocol can be accessed, if available	n/a
Funding	25	Sources of funding and other support (such as supply of drugs), role of funders	11
	26	Ethical approval or approval by research review committee, confirmed with reference number	5

Citation: Eldridge SM, Chan CL, Campbell MJ, Bond CM, Hopewell S, Thabane L, et al. CONSORT 2010 statement: extension to randomised pilot and feasibility trials. BMJ. 2016;355. This is an Open Access article distributed in accordance with the terms of the Creative Commons Attribution (CC BY 3.0) license (http://creativecommons.org/licenses/by/3.0/), which permits others to distribute, remix, adapt and build upon this work, for commercial use, provided the original work is properly cited.

*We strongly recommend reading this statement in conjunction with the CONSORT 2010, extension to randomised pilot and feasibility trials, Explanation and Elaboration for important clarifications on all the items. If relevant, we also recommend reading CONSORT extensions for cluster randomised trials, non-inferiority and equivalence trials, non-pharmacological treatments, herbal interventions, and pragmatic trials. Additional extensions are forthcoming: for those and for up-to-date references relevant to this checklist, see www.consort-statement.org.
